# Near-infrared cholangiography can increase the chance of success in laparoscopic approaches to common bile duct stones, even with previous abdominal surgery

**DOI:** 10.1186/s12893-023-02103-6

**Published:** 2023-07-15

**Authors:** Wei-Juo Tzeng, Yu-Hung Lin, Teng-Yuan Hou, Shih‑Min Yin, Yu-Cheng Lin, Yueh-Wei Liu, Yu-Yin Liu, Wei-Feng Li, Chih-Chi Wang, Jacques Marescaux, Michele Diana

**Affiliations:** 1 Division of General Surgery, Department of Surgery, Kaohsiung Chang Gung Memorial Hospital and Chang Gung University College of Medicine, No.123, Dapi Rd., Niaosong Dist., Kaohsiung City, Taiwan; 2 IRCAD, Research Institute Against Digestive Cancer, Strasbourg, France; 3 ICube Lab, Photonics for Health, University of Strasbourg, Strasbourg, France; 4 Department of Surgery, University Hospital of Strasbourg, Strasbourg, France

**Keywords:** Fluorescence-guided surgery, Indocyanine green, Laparoscopic common bile duct stone exploration, Near-infrared cholangiography, Common bile duct stone

## Abstract

**Background:**

The treatment of common bile duct (CBD) stones with minimally invasive surgery (MIS) is more technical demanding than laparoscopic cholecystectomy (LC), especially in patients with history of previous abdominal surgery, cholangitis or cholecystitis. Near-infrared (NIR) cholangiography via systemic or biliary tree administration of indocyanine green (ICG), which enhances the visualization of the biliary tree anatomy, may increase the reassurance of CBD localization. The aim of this study was to identify the benefit of near-infrared cholangiography for laparoscopic common bile duct exploration (LCBDE).

**Methods:**

Three groups of CBD stone patients were included in this retrospective study depending on the surgical methods: 1) open choledocholithotomy (OCC), 2) laparoscopic choledocholithotomy (LCC), and 3) near-infrared cholangiography-assisted laparoscopic choledocholithotomy (NIR-CC). For the NIR-CC group, either 3 ml (concentration: 2.5 mg/mL) of ICG were intravenously administered or 10 ml (concentration: 0.125 mg/mL) of ICG were injected directly into the biliary tree. The enhancement rate of the cystic duct (CD), CBD, the upper and lower margin of the CBD were compared using white light image.

**Results:**

A total of 187 patients with a mean age of 68.3 years were included (OCC, *n* = 56; LCC, *n* = 110; NIR-CC, *n* = 21). The rate of previous abdominal surgery was significantly lower in the LCC group. The conversion rate was similar between the LCC and the NIR CC groups (*p* = 0.746). The postoperative hospital stay was significantly longer in the OCC group. No differences in morbidity and mortality were found between the three groups. In the NIR-CC group, the localization of CBD was as high as 85% compared to 24% with white light imaging.

**Conclusions:**

Near-infrared cholangiography helps increase the chance of success in minimally invasive approaches to CBD stones even in patients with previous abdominal surgeries, without increasing the rate of conversion.

## Background

Common bile duct (CBD) stones occur in 10 to 20% patients with gallstones [1]. CBD stones can be classified according to the location of their origin: (1) primary bile duct stones, forming initially in the bile ducts; (2) secondary to gallbladder stones, originating in the gallbladder and passing into the bile ducts; and (3) secondary to or coexisting with intrahepatic bile duct stones [2]. The comparison between single stage laparoscopic common bile duct exploration (LCBDE) and endoscopic retrograde cholangiopancreatography (ERCP) plus laparoscopic cholecystectomy remains inconclusive [3], although the former requires fewer procedures and has similar outcomes [4].

In the past, LCBDE was indicated when endoscopic stone extraction failed [5, 6]. As laparoscopic experience increases and the suturing techniques becomes more mature, the rate of LCBDE increased, but conversion to laparotomy is still common due to biliary anatomical variations, inflammatory conditions, or adhesions caused by previous abdominal surgeries [7]. For example, the conversion rate of LCBDE was relatively higher in patients with previous gastrectomy [8]. An initial laparotomy for CBD stone extraction tended to be chosen for patients with previous gastrectomy.

Indocyanine green (ICG), which can be illuminated by a near-infrared light source, can help to identify the CBD by real-time fluorescent signal during surgery to prevent CBD injury. Recently, fluorescent cholangiography using an intravenous or biliary tree injection of ICG, and near-infrared optimized laparoscope has been widely applied to cholecystectomy to visualize the biliary tree [9, 10]. Furthermore, current research has mostly focused on the benefits of fluorescent cholangiography via systemic injection (intravenous, IV) of ICG. Our previous trial focused on the fluorescent effect via direct injection of diluted ICG [11, 12] into the biliary tract and found it was beneficial to identify the bile duct without the need of systemic injection. ICG biliary injection provides real-time fluorescent imaging without the interference of liver background noise compared to the systemic injection of ICG that requires 30–40 min before the signal can be visualized. NIR cholangiography has been applied to laparoscopic cholecystectomy and improves the visualization of the biliary anatomy, resulting in decreased CBD injury and mortality [13]. So far, the benefit of NIR cholangiography in CBD stone treatment has not been well-described yet.

For CBD stones-related obstructive jaundice, preoperative drainage was sometimes indicated for biliary tree infection. Internal drainage, performed with endoscopic retrograde biliary drainage (ERBD) and external drainage, performed with percutaneous transhepatic common hepatic duct drainage (PTCD) or percutaneous transhepatic gallbladder drainage (PTGBD), could be chosen according to the patient’s condition [14–16]. As a result, fluorescent cholangiography can be performed via systemic injection or biliary injection of ICG. The purpose of this study was to evaluate the feasibility and usefulness of fluorescent cholangiography in patients with CBD stones.

## Materials and methods

### Patient selection

Between 2016 and 2021, 187 patients with CBD stones treated surgically were included in this retrospective study at the Department of Surgery in the Kaohsiung Chang Gung Memorial Hospital, Taiwan. All the patient received the computed tomogram (CT) scan for confirming the CBD stone. The patients were divided into three groups: open choledocholithotomy (OCC, *n* = 56); laparoscopic choledocholithotomy (LCC, *n* = 110); and near-infrared cholangiography-assisted laparoscopic choledocholithotomy (NIR-CC, *n* = 21). Patient characteristics were recorded, including age, gender, BMI, preoperative biochemical data, preoperative drainage method such as ERBD, PTCD, and PTGBD, and postoperative outcomes. The complication was classified according to Clavien-Dindo classification [17]. We defined grade 1,2 and 3a as minor complications; and grade 3b, 4a, 4b and 5 were recorded as major complication. The study was approved by the Institutional Review Board of the Chang Gung Memorial Hospital (No.202200304B0).

### NIR Cholangiography Procedures (NIR-CC)

Dosage of ICG to illuminate extrahepatic biliary tract:

Patients undergoing preoperative external drainage, such as percutaneous transhepatic gallbladder drainage (PTGBD) or percutaneous transhepatic bile duct drainage (PTCD), would receive intrabiliary ICG administration. The PTGBD or PTCD tube was prepared via an aseptic method and the gallbladder bile was removed as much as possible. Next, 0.5 ml 2.5 mg/mL concentrations of ICG (DIAGNOGREEN® 25 mg/10 mL, Taiwan) were diluted with 9.5 ml of distilled water with final concentration of 0.125 mg/mL. A total of 10 ml of diluted ICG was then injected into the gallbladder through the PTGBD tube or IHD through the PTCD tube.

For patients without external drainage tubes, IV systemic ICG administration was performed: 3 ml 2.5 mg/mL concentrations of ICG were injected into the peripheral venous system 30 min before surgery.

The D-Light, a near-infrared optimized laparoscope (Karl Storz, Tuttlingen, Germany) with or without Spectra A mode, was used to detect the ICG fluorescence signal arising from the cystic duct (CD), CBD, upper margin of the CBD, and lower margin of the CBD before and during the operation.

### White Light (WL) / fluorescent identification method

Because the surgical technique was dynamic according to the anatomical condition, the dynamic recording method was used for white light and fluorescent identification. During the operation, the transition between white light and fluorescent imaging was performed frequently. If the fluorescent signal enhanced the CBD but the CBD still could not be identified using white light imaging simultaneously, we recorded white light CBD (-) and fluorescent CBD ( +). If the CBD could be identified with both white light and fluorescent imaging, we recorded white light CBD ( +) and fluorescent CBD ( +). If the CBD could be identified with white light imaging but not with fluorescent imaging for any reason, such as insufficient time after ICG injection or thickness of peri-CBD adipose tissue, we recorded white light CBD ( +) and fluorescent CBD (-).

### Near-infrared laparoscopic choledocholithotomy

**Fig. 1:**
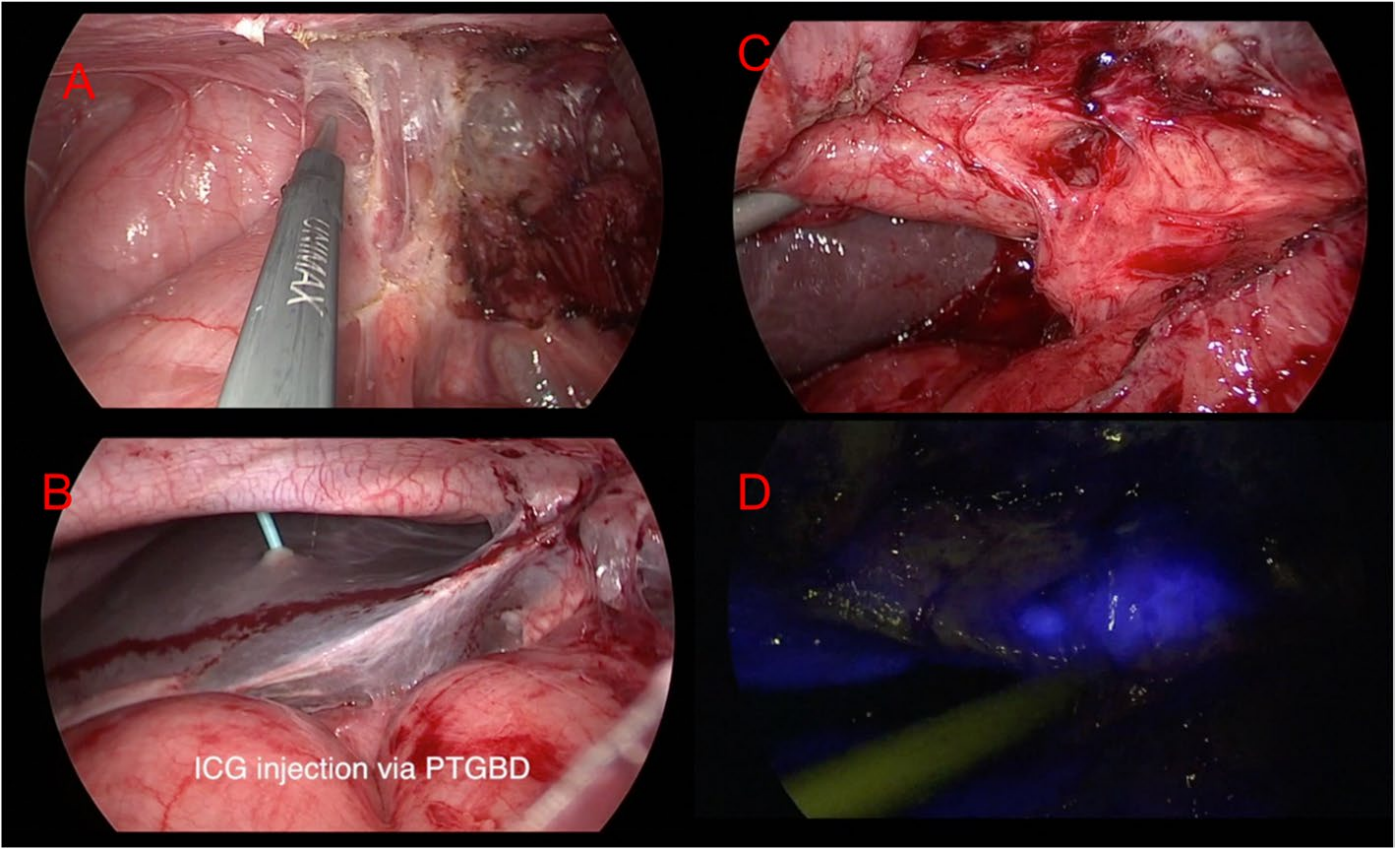
Fluorescent and white light imaging of biliary tree before hilum dissection. **A** Grade IV adhesion due to previous gastric surgery. **B** The ICG was injected via percutaneous transhepatic gallbladder drainage (PTGBD). **C** Identification recording of the biliary tree using white light imaging: CD (-), CBD (-). **D** Fluorescent enhancement recording of the biliary tree via NIR imaging CD ( +), CBD ( +)

**Fig. 2:**
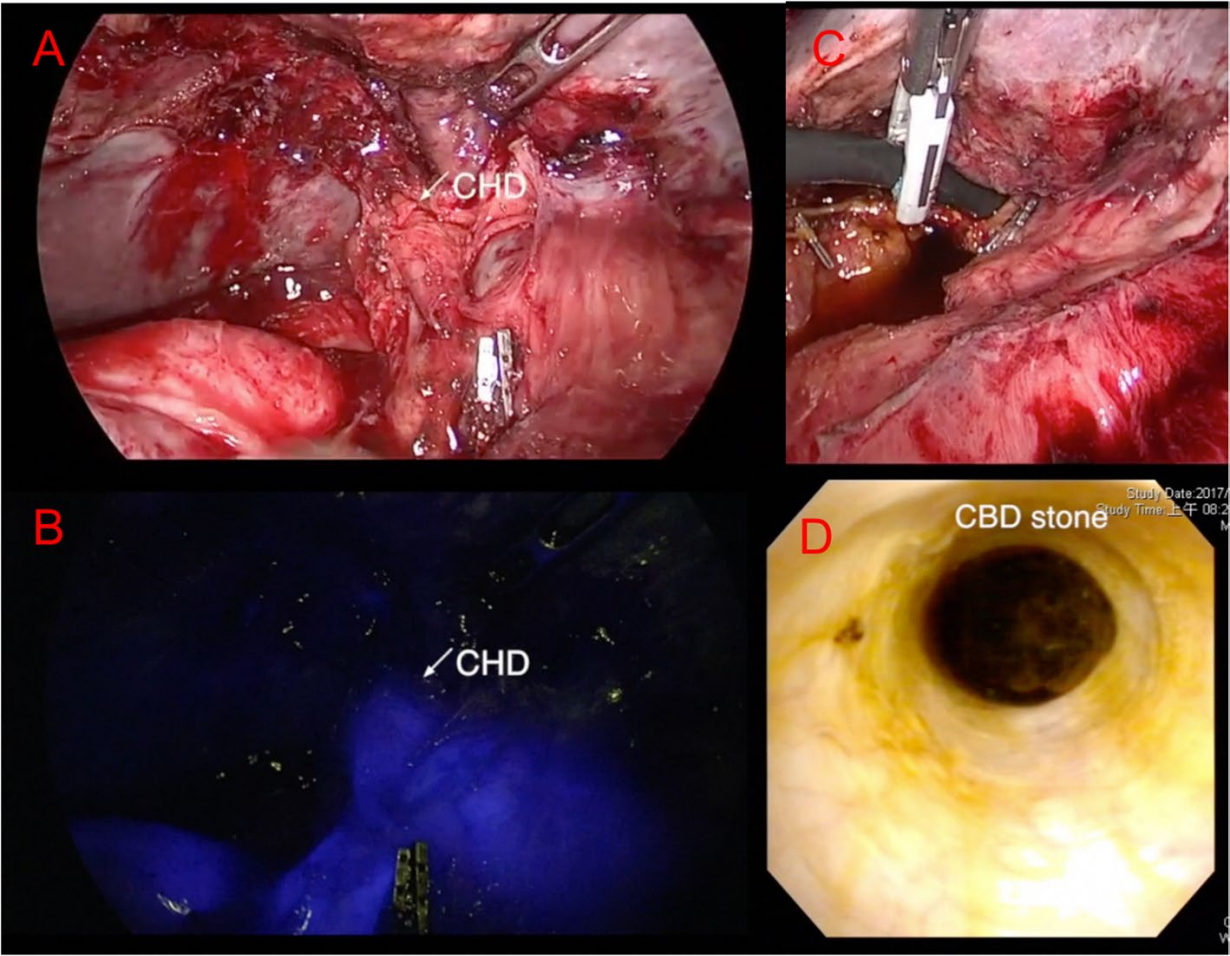
Fluorescent and white light imaging of biliary tree before choledochotomy. **A** Before choledochotomy, identification recording of the biliary tree using white light imaging: CBD upper margin (-), lower margin (-). **B** Fluorescent enhancement recording of the biliary tree using NIR image: CBD upper margin ( +), lower margin ( +). **C** Choledochoscope insertion into CBD. **D** CBD stone detection with choledochoscope

Laparoscopic choledocholithotomy was performed using the standard 4-trocar method. Peri-gallbladder adhesiolysis was firstly performed to expose the gallbladder or hepatic hilum (Fig. 1A, B) and kept dissection up to the exposure of the CBD using white light or fluorescent imaging, and the percentage of visualization of the extrahepatic bile duct system could be recorded (Fig. 1C, D). The visualization percentages of the upper and lower margins of the CBD were recorded again before choledochotomy (Fig. 2A, B). The CBD was opened with or without cholecystectomy. The choledochoscope was inserted into the CBD and stones were removed via a Dormia basket. After the operator evaluate the risk of the recurrent CBD stone, the thickness of the CBD wall and the fragile of the CBD, the choledochotomy was closed with or without a T tube (Fig. 2C, D). After the operation, we also reviewed the surgical videos and categorized into 4 grades according to Nassar operative difficulties [18].

### Statistical analysis

Statistics were performed using SPSS® Statistics, version 25. The one-way ANOVA was performed for post-operation admission days, operation time, pre-operation data. Then, we also use the Pearson's chi-squared test and Fisher’s exact test for comparison of ERCP, PTGBD arrangement, abdominal operation history. Because the all the converted cases were placed in NIR-CC and LCC groups. The conversion rate comparison was only including these two groups. The Fisher’s exact test was used to calculate NIR accuracy and compare with WL observation (*P* < 0.05 was considered statistically significant).

## Results

A total of 187 patients were included. Demographic data is displayed in Table 1. The female-to-male ratio was 0.8 (84/103) in all patients. The average BMI was 24.32 kg/m^2^. No significant differences were found in the preoperative serum biochemistry parameters between the three groups. Regarding postoperative outcomes, mean hospital stay was significantly higher in OCC and NIR-CC groups (OCC:16 days, LCC: 8 days, NIR-CC: 12.8 days, *p* = 0.0001). In the LCC groups, the patients were with significantly lower rate of previous abdominal surgery compared to the NIR-CC group (OCC: 57.1%, LCC: 11.8%, NIR-CC: 47%, *p* = 0.0001). The conversion rate was similar in the LCC and NIR-CC groups (LCC: 15.5%, NIR-CC:19%, *p* = 0.746) There was no difference in the morbidity and mortality between the three groups.

**Table 1:** Choledocholithotomy patient demographic data

	OCC	LCC	NIR-CC	*P* value
Patients	56	110	21	
Age (year)	69 (33–95)	68 (24–98)	70.8 (46–92)	0.774
Gender				
Female (%)	25 (44.6%)	52 (47.3%)	7 (33%)	0.36
Male (%)	31 (55.4%)	58 (52.7%)	14 (67%)	
BMI(kg/m^2^)	24.13 (17.2–34.9)	24.27 (13.6–40.4)	25.17 (16.1–34.7)	0.41
CRP(mg/L)	33.7 (0.5–380)	37.2 (0.2–359)	16.4 (0.4–57.4)	0.548
Alkaline Phosphatase (U/L)	188.8 (52–742)	160.4 (38–677)	196.5 (51–693)	0.349
WBC(1000/μL)	7.5 (3.4–25)	7.4 (3–19.8)	6.43 (3.9–12.6)	0.471
Bilirubin T (mg/dL)	1.77 (0.2–15.3)	1.61 (0.2–13.8)	1.77 (0.3–8.8)	0.859
Preoperative drainage				
ERBD (%)	19 (33.9%)	52 (47.3%)	11 (52.4%)	0.216
PTCD (%)	4 (7.1%)	4 (3.6%)	4 (19%)	0.041
PTGBD (%)	7 (12.5%)	18 (16.4%)	6 (28.6%)	0.195
T tube placement (%)	40 (71.4%)	47 (42.7%)	17 (81%)	0.043
Abdominal surgical history (%)	32 (57.1%)	13 (11.8%)	10 (47%)	0.0001
Operative time(minutes)	181 (74–450)	237 (108–580)	289 (163–454)	0.0001
Conversion (%)^*^	-	17 (15.5%)	4 (19%)	0.746^*^
Major complications (%)	0 (0%)	3 (2.7%)	0 (0%)	0.404
Minor complications (%)	8 (14.3%)	21 (19.1%)	5 (24%)	0.948
PHOS (days)	16 (3–179)	8 (2–64)	12.8 (3–16)	0.0001

*PHOS* Postoperative hospital stays

^*^Fisher exact test between LCC and NIR-CC

In the previous analysis, the abdominal surgical history, operation time, postoperative hospital stays (PHOS) were significantly different between the three groups. The post hoc test showed that, in PHOS, the OCC group had significantly longer duration compared to NIR-CC and LCC. Regarding operation time, the OCC group had significantly shorter operation time compared to the other two groups. As for history of previous abdominal surgery, the LCC had significantly lower incidence compared to OCC and NIR-CC.

Regarding the history of previous abdominal surgery, 13 LCC patients had abdominal surgery history; and most of them underwent laparoscopic cholecystectomy (5/13) or laparoscopic herniorrhaphy (8/13). Ten patients had a history of previous abdominal surgery in the NIRCC group (5 subtotal gastrectomy, two hemicolectomy, one open choledocholithotomies, and two laparoscopic cholecystectomies). The conversion rate was 15.5% (17/110) in the LCC group, mostly due to severe adhesions and unclear identification of the biliary tree. The conversion rate was 19% (4/21) in the NIRCC group; two conversions were due to voluminous stones, which could not be removed laparoscopically, one was because of iatrogenic colon perforation, and another one was due to the incapability to visualize CBD.

In the NIR-CC group, no patient experienced procedure failure due to ICG leak. There were 15 (71%) patients with high-grade surgical difficulty (Table 2). For the first stage (dissection until the identification of the CBD with white light or fluorescent imaging), the rates of visualization of the cystic duct were 22% for white light, and 55% for fluorescent imaging in 18 patients; 24 and 85% for the common bile duct in 21 patients. For the second stage (dissection to choledochotomy), the rates of visualization of the upper margin of the CBD were 19 and 62% respectively; and 29 and 57% for the lower margin of the CBD (Table 2).

**Table 2:** White/NIR visualization in NIR-CC

ICG injection route	No	Difficulty^a^ I, II/III-V	White/NIR visualization			
			CD^b^	CBD	Upper margin	Lower margin
IV	11	4/7	3/4	5/9	4/7	5/7
PTCD	4	1/3	0/2	0/4	0/3	0/2
PTGBD	6	1/5	1/4	0/5	0/3	1/3
Total	21	6/15 (28.5%/71%)	4/10 (22%/55%) (P:0.08)	5/18 (24%/85%) (P: 0.001)	4/13 (19%/62%) (P:0.012)	6/12 (29%/57%) (P:0.07)

*ICG* Indocyanine green, *IV* Intravenous, *NIR* Near Infrared, *PTCD* Percutaneous Transhepatic Cholangiography and Drainage, *PTGBD* Percutaneous Transhepatic Gallbladder Drainage

^a^Nassar operative difficulties or Adhesion score

^b^Total of 18 cases, 3 patients had received cholecystectomy before

## Discussion

Minimally invasive procedure is currently the gold standard for cholecystectomy. However, for common bile duct stones, it is still a matter for debate. An increasing number of studies describe the benefits of one-stage treatment for gallstones combined with CBD stones using minimally invasive surgery instead of ERCP combined with LC [19, 20]. For patients with a history of previous gastrectomy with gastrojejunostomy, ERCP and LCBDE were both possible solutions for CBD stones [21]. However, the difficulty of ERCP increases with preceding Billroth II reconstruction and multiple/large CBD stones removal [22]. There are two key challenges related to CBD stone surgery, namely anatomical localization, and laparoscopic skills. In recent years, many articles described the technique and the results of LCBDE, but relatively fewer researches focused on how to improve anatomical localization, especially in cases of inflammation or with previous abdominal surgeries. Generally, LCBDE would only be considered if the CBD stone is relatively simple, and the patients have not previously undergone complex abdominal surgeries (i.e., stomach, ascending colon, or liver surgeries). Because the extensive adhesion due to previous complex abdominal surgeries may hinder the identification of biliary structures. With the assistance of near-infrared fluorescent laparoscopy, the identification of the biliary tract becomes more feasible in the patients with dense adhesion, so the success rate of LCBDE could increase.

The near-infrared fluorescence was widely used in the different field. In the previous study, this was used for lymph node dissection during laparoscopic gastrectomy [23], pancreatic and periampullary cancer [24] or gallstone [25, 26]. But there were only few articles about the application of NIR in LCC. With our experience in applying near-infrared fluorescence on laparoscopic cholecystectomy, we wanted to extend the application of near-infrared fluorescence [12]. As described in our previous article, two methods can be used for the illumination of the CBD, namely intrabiliary ICG administration or systemic ICG administration. The details of their benefits have been described in our previous article [11, 12]. In patients with obstructive jaundice, internal drainage such as ERBD or external drainage such as PTGBD or PTCD may be performed preoperatively. In our study, we injected ICG into external drain tubes in patients with PTGBD or PTCD, and injected ICG to the systemic circulation in patients without biliary drainage.

In our result, the rate of previous surgical history in the LCC group was only 11.8% and was significantly higher, 47.6%, in the NIR-CC group. However, the conversion and complication rates were comparable between LCC and NIR-CC groups. With higher incidence of preceding abdominal operations in NIR-CC group, the surgical complexity should raise substantially due to possible extensive adhesion. Nonetheless, with the help of NIR, the rate of converting to laparotomy was not higher than the LCC group. This result indicates that near-infrared fluorescence is very helpful in aiding the visualization of the biliary structure to greatly improve the success rate of finishing the surgery with minimally invasive approach.

Most conversions in the NIR-CC group were not related to anatomical localization. Only one patient was converted to open surgery due to an unvisualized CBD. Two other conversions were due to an extremely large stone impaction and iatrogenic colon injury. The most common reason for conversion in the LCC group was related to unclear anatomy or adhesions. In the NIR-CC group, the success rate of fluorescent enhancement of the CBD was 85% compared to 24% in white light imaging. Fluorescent imaging can prevent accidental CBD injuries. It can also show the border of the CBD and prevent erroneous positioning of the choledochotomy. This erroneous positioning can occur in cases of unclear CBD anatomy and can increase the difficulty of stone removal and the closure of the CBD.

In our series, some selection bias may exist. The NIR scope was introduced as the new technique after the evidence of our trial for laparoscopic cholecystectomy [12]. Difficult cases or patients having undergone previous abdominal surgery would be advised to undergo this image-guided surgery. As a result, the percentage of previous abdominal surgery was higher in the NIR-CC group. The difficulty of surgery was also increased and grade III-V adhesions [27] or Nassar grade III-IV adhesions were about 71% (15/21) in the NIR-CC group, but the conversion rate was not increased compared to the LCC group. The visualization rate of the CBD, upper margin and lower margin of CBD imaging were 85, 62, and 57% respectively. By adding fluorescent imaging, the successful rate of minimally invasive surgeries for CBD stones will increase and surgeons will feel more confident when faced with difficult cases.

The major differences between intrabiliary and systemic ICG administration were fluorescence signal onset and background noise. Intrabiliary administration can provide real-time imaging without background fluorescent noise. However, perforation of the gallbladder with intraoperative bile leakage can lead to failure of fluorescent imaging. Regarding systemic administration, the timing of the injection was crucial. It took 30 to 40 min for the ICG to get into the bile and generate fluorescent signals. It also yields less clear fluorescent imaging because of the background noise. The common problem of those two methods is that the fluorescent signal could not be retrieved if the CBD wall was thicker than 0.5 cm.

## Conclusions

In conclusion, near-infrared cholangiography can increase the chance of success in minimally invasive approaches to common bile duct stones, especially in patients with history of previous abdominal surgery.

## Data Availability

The datasets used and/or analyzed during the current study are available from the corresponding author on reasonable request.

## Abbreviations

CBD: Common bile duct
MIS: Minimally invasive surgery
LC: Laparoscopic cholecystectomy
NIR: Near-infrared
ICG: Indocyanine green
LCBDE: Laparoscopic common bile duct exploration
CT: Computed tomogram
OCC: Open choledocholithotomy
LCC: Laparoscopic choledocholithotomy
NIR-CC: Near-infrared cholangiography-assisted laparoscopic choledocholithotomy
CD: Cystic duct
ERCP: Endoscopic retrograde cholangiopancreatography
ERBD: Endoscopic retrograde biliary drainage
PTCD: Percutaneous transhepatic common hepatic duct drainage
PTGBD: Percutaneous transhepatic gallbladder drainage
WL: White Light
PHOS: Postoperative hospital stays
